# Patient self-inflicted lung injury an important phenomenon

**DOI:** 10.1097/MCC.0000000000001348

**Published:** 2025-12-08

**Authors:** Eleonora Balzani, Glasiele C. Alcala, Giacomo Bellani, Antonio Pesenti

**Affiliations:** a Centre for Medical Sciences-CISMed, University of Trento, Trento, Italy; b Department of Anesthesia, Critical Care and Pain Medicine, Massachusetts General Hospital, Boston, Massachusetts, USA; c Anesthesia and Intensive Care 1, Santa Chiara Hospital, APSS, Trento; d Department of Pathophysiology and Transplantation, Università di Milano, Milan, Italy

**Keywords:** acute hypoxemic respiratory failure, diaphragm., mechanical ventilation, patient self-inflicted lung injury, respiratory drive

## Abstract

**Purpose of review:**

Mechanical ventilation is essential in acute hypoxemic respiratory failure (AHRF), yet excessive respiratory drive and inspiratory effort may aggravate injury, a phenomenon termed patient self-inflicted lung injury (P-SILI). This review summarizes mechanistic insights, preclinical and clinical evidence, and current strategies to prevent P-SILI while preserving diaphragmatic function.

**Recent findings:**

Preclinical experimental studies show that vigorous inspiratory efforts amplify pleural pressure swings, regional overdistension, pendelluft, and inflammation, with damage involving both lung and diaphragm. positive end-expiratory pressure (PEEP) and continuous positive airway pressure (CPAP) can homogenize ventilation, reduce strain-rate, and protect diaphragmatic mechanics, whereas uncontrolled effort worsens outcomes. Clinical investigations confirm that high drive and effort increase total lung stress despite protective tidal volumes and are linked to mortality, ventilator dependence, and complications such as pneumomediastinum. Emerging approaches include titrated pressure support and sedation and ventilatory assistance, neuromuscular blockade, phrenic nerve block, pharmacological drive modulation, prone positioning, and extracorporeal CO_2_ removal. Strategies aimed at preserving diaphragm activity, such as electrical phrenic stimulation or inspiratory muscle training, further broaden protective options.

**Summary:**

P-SILI arises when excessive inspiratory effort translates into injurious lung and diaphragm stress. Preventive strategies should not abolish but shape effort, integrating ventilatory settings, sedation, and drive-modulating interventions across the continuum from the acute phase to weaning and rehabilitation.

## INTRODUCTION

Mechanical ventilation remains a cornerstone in the management of acute respiratory failure (ARF). Over the past two decades, the recognition of ventilator-induced lung injury (VILI) has reshaped critical care, making lung-protective ventilation the standard approach and definitively abandoning the historical practice of high-volume, high-pressure ventilation.

While primarily conceived as a supportive intervention, mechanical ventilation is increasingly viewed as an active tool to limit the progression of lung damage. In severe ARF, excessive respiratory drive and inspiratory effort can aggravate injury, a phenomenon described as patient self-inflicted lung injury (P-SILI). This concept has introduced the notion that, under certain circumstances, controlling or even suppressing spontaneous respiratory activity may protect the lung, thereby conferring a protective role to controlled mechanical ventilation beyond simple support [1].

Recent experimental and clinical studies have explored the mechanisms through which excessive effort and drive – essentially the patient-generated counterpart of injurious ventilation – contribute to pulmonary and diaphragmatic injury. These investigations highlight the potential benefits of strategies aimed at tempering or suppressing strong inspiratory efforts to achieve both lung and diaphragm protection.

This review will examine current insights into the pathophysiology of P-SILI, summarize the most relevant clinical evidence, and discuss established and emerging approaches to modulate respiratory drive and effort, with particular attention to interventions designed to preserve diaphragmatic function throughout the course of ARF. 

**Box 1:**
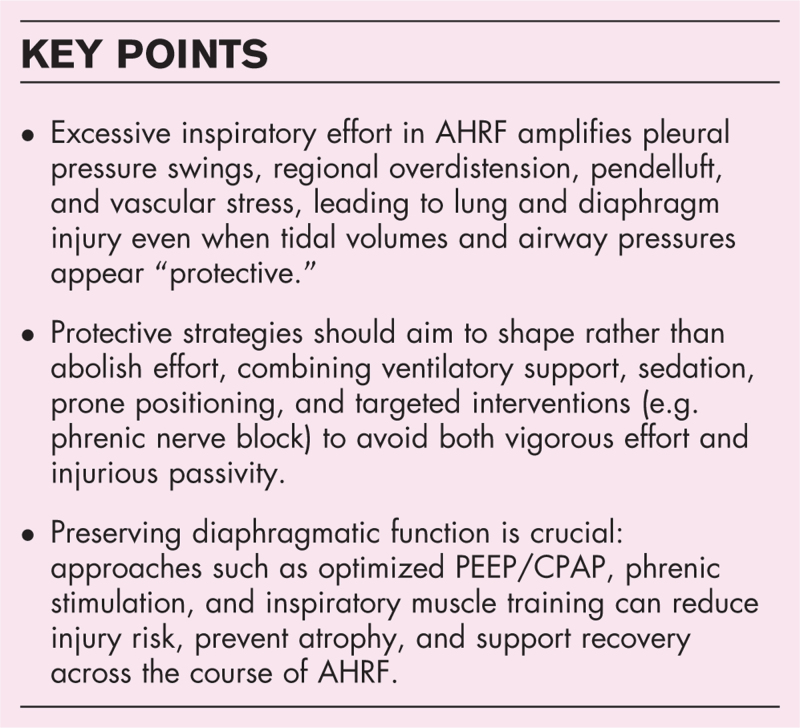
no caption available

## MECHANISMS OF PATIENT SELF-INFLICTED LUNG INJURY AND PRECLINICAL EVIDENCE

Acute hypoxemic respiratory failure (AHRF) poses a dual challenge: alleviating dyspnea and hypoxemia while preventing P-SILI and diaphragm damage.

Vigorous inspiratory efforts generate large negative pleural pressure swings, amplifying regional transpulmonary stresses and intraparenchymal gas shifts. This creates intra-alveolar pendelluft, which can overdistend dependent lung regions while simultaneously deflating nondependent areas, even when global tidal volume and airway pressures appear well tolerated [2].

Low positive end-expiratory pressure (PEEP) with preserved effort concentrates stress/strain and inflammation in dependent lung, whereas sufficient PEEP can render spontaneous effort noninjurious by homogenizing ventilation and blunting pleural pressure gradients [3,4].

Animal data confirm that the intensity of effort critically determines the trajectory of injury: in a porcine model of surfactant depletion, spontaneous breathing under minimal support worsened oxygenation, increased esophageal pressure swings, amplified ventilation heterogeneity measured by EIT, and produced more severe histologic damage compared with early controlled ventilation [5]. Likewise, in a murine model of acute respiratory distress syndrome (ARDS) induced by LPS, resistive breathing exacerbated hypoxemia and inflammation, downregulating Piezo1, a mechanosensitive channel implicated in ventilator-induced injury, underscoring the combined burden on lung and diaphragm [6]. By contrast, in a severe ARDS–ECMO porcine model, low-intensity rapid shallow breathing generated small tidal volumes and low ΔPes and did not increase histologic injury relative to near-apneic ventilation, suggesting that effort becomes harmful only when vigorous [7].

In parallel, diaphragm-centric data show that strenuous, unsupported breathing loads the muscle and accelerates injury; importantly, noninvasive continuous positive airway pressure (CPAP) attenuates in mice and pigs both lung and diaphragm damage in experimental lung injury, positioning this as a truly integrated protective strategy [8^▪▪^,^9^]. Two newer insights sharpen the frame. First, injury progression is set not only by amplitude of tissue deformation (strain) but also by how fast deformation occurs (strain rate), with recent work – including micro-computed tomography (μCT) analysis – demonstrating that CPAP reduced both inspiratory and expiratory strain-rate and improved the velocity of diaphragmatic relaxation [8^▪▪^,^10^].

Importantly, recent in-silico models have emphasized that PEEP may act as a double-edged sword. Digital-twin cardiopulmonary simulations show that successful noninvasive ventilation coincides with approximately 57% reduction in the patient's driving pressure, reflecting unloading of inspiratory effort; if effort is not reduced, oxygenation may improve while lung stress remains elevated, predicting NIV failure [11]. These integrated data argue that protective strategies must target effort control and expiratory mechanics in addition to oxygenation.

Second, patient-ventilator interface and flow physics matter: computational and bench data show that the endotracheal-tube jet and Venturi effects can sustain intrathoracic flow deflection and pendelluft despite higher inlet pressure, explaining why “just turning up support” will not fully solve effort-driven heterogeneity without reducing patient effort [12]. Expiratory mechanics are not a side note, they belong to the strategic plan in P-SILI and should be measured and managed [8^▪▪^].

## CLINICAL EVIDENCE OF PATIENT SELF-INFLICTED LUNG INJURY

P-SILI occurs when excessive inspiratory effort (ΔPes) is transmitted to injured lungs, with damage typically concentrating in dependent (dorsal) regions. Vigorous spontaneous effort exacerbates injury through four main pathways: global and regional overdistension, increased pulmonary perfusion, negative-pressure pulmonary edema from elevated transmural vascular pressures [13], and pendelluft.

**FIGURE 1:**
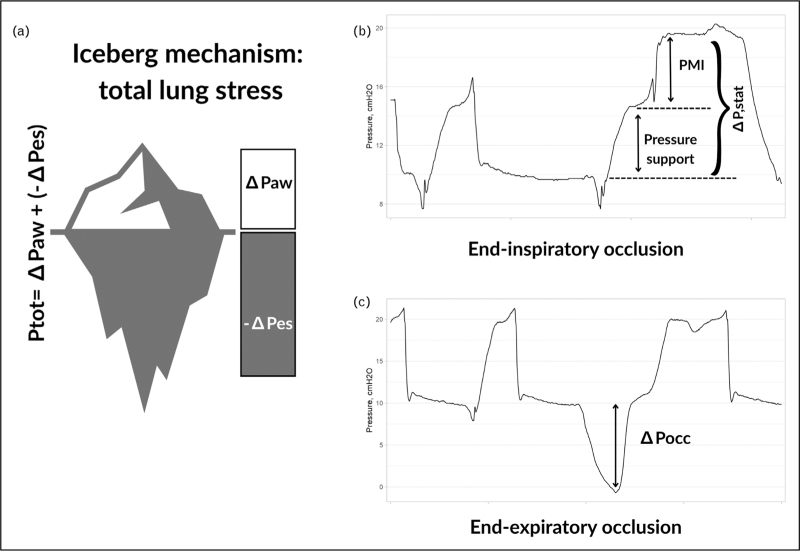
The “iceberg” mechanism of total lung stress in patient self-inflicted lung injury (P-SILI). Panel A illustrates the “iceberg” concept, where the apparent stress generated by airway pressure (ΔPaw, white bar) represents only the visible portion, while the hidden component generated by inspiratory muscle effort (−ΔPes, gray bar) adds substantially to total lung stress (Ptot = ΔPaw + (−ΔPes). Panel B shows representative airway pressure tracings during end inspiratory occlusion. The total static driving pressure (ΔPstat) results from the sum of pressure support and patient effort, with the pressure muscle index (PMI) reflecting inspiratory muscle contribution. Panel C depicts an end expiratory occlusion maneuver, where the deflection in esophageal pressure (ΔPocc) quantifies patient inspiratory effort. ΔPaw, airway pressure swing; ΔPes, esophageal pressure swing; ΔPocc, pressure occlusion maneuver; ΔPstat, static driving pressure; PMI, pressure muscle index; Ptot, total lung stress.

Large prognostic studies highlight the clinical relevance of respiratory effort. Elevated P0.1, higher inspiratory effort measured by PMI or Pmus, and increased driving pressure during assisted ventilation have all been associated to lower ICU survival and prolonged ventilation [14,15^▪▪^,^16^] (Fig. 1).

Physiological investigations provide mechanistic insights. Zhang *et al.* [17] demonstrated that real-time monitoring of esophageal pressure, dynamic driving pressure, and regional ventilation (via electrical impedance tomography) can uncover injurious efforts not apparent from tidal volume or plateau pressure alone. The ICEBERG study [15^▪▪^] elegantly proved this concept: patient effort and ventilator pressure add up like the visible and hidden parts of an iceberg, what is seen at the airway represents only a fraction of the total load, while the concealed negative pressure generated by inspiratory muscles may turn apparently “protective” ventilation into injurious breathing. In 298 patients with AHRF, nonsurvivors exhibited higher static and dynamic driving pressures despite similar tidal volumes, peak pressures, and ΔPocc, underscoring that outcomes are determined less by effort itself than by its translation into total lung stress [15^▪▪^].

Consistently, Telias *et al.* [18^▪^] showed that greater inspiratory effort (Pmus) was associated with higher transalveolar driving pressure, larger tidal volumes, and lower inspiratory alveolar pressures relative to PEEP. These physiological alterations had prognostic implications: higher stress predicted loss of compliance, while reduced inspiratory alveolar pressure was linked to impaired oxygenation [18^▪^].

Smaller clinical reports also support a causal role of excessive effort. Watanabe *et al.* [20] described cases of pneumomediastinum resolving after suppression of spontaneous breathing efforts with neuromuscular blockade, with CT evidence of the Macklin effect, a hallmark of alveolar rupture caused by large transpulmonary pressure swings [19].

This phenomenon appears consistent across ventilatory modes. In a prospective study, Serrano *et al.* found that respiratory drive and inspiratory effort remained elevated across PSV, APRV, and BiLevel ventilatory modes, regardless of underlying elastance. These results suggest that no spontaneous mode reliably attenuates excessive effort, reinforcing the view that uncontrolled drive, rather than the ventilatory mode itself, sustains the risk of P-SILI [21].

Pendelluft has also been proposed as a distinct mechanism of regional injury. Cornejo *et al.* [22^▪^] observed high-magnitude pendelluft (≥10% of tidal volume (VT) redistributed between regions) in 37.5% of ARDS patients transitioning from controlled to partial support, accompanied by significant increases in IL-8, IL-18, and Caspase-1 within 4 h. Other studies, however, report less consistent results. Its occurrence and magnitude appears to depend not only on inspiratory effort but also on lung heterogeneity, compliance gradients, collapse, time constants, and PEEP distribution [23–25]. Moreover, thresholds for defining clinically relevant pendelluft vary widely (≥10% to ≥25% of VT), limiting comparability. Clinically, high levels of pendelluft have been linked to prolonged ventilation, longer ICU stay, and frequent weaning failure [22^▪^,^23^]. Whether it represents an independent mechanism of injury or simply reflects the interplay between effort and regional mechanics remains debated.

Taken together, experimental and clinical evidence converge on a unifying concept: P-SILI arises when excessive inspiratory effort translates into injurious global or regional stress. This may manifest as alveolar rupture (Macklin effect), vascular stress and edema, or heterogeneous strain amplified by pendelluft. Because these mechanisms are not captured by tidal volume or plateau pressure alone, modulation of inspiratory effort – quantified by ΔPes and expressed as total driving pressure – emerges as a central therapeutic target for lung-protective strategies.

## STRATEGIES TO AVOID PATIENT SELF-INFLICTED LUNG INJURY

### Shaping effort: the balance of sedation and assistance

At this stage of the discussion on P-SILI, a more fundamental question emerges: does excessive inspiratory effort represent a cause, a consequence, or both in the development of lung injury? Diaphragmatic dysfunction epitomizes this duality, acting simultaneously as a driver and as an outcome of P-SILI. On the one hand, increased inspiratory effort – often triggered by hypoxemia and reduced lung compliance – generates high transpulmonary pressures and mechanical stress, aggravating alveolar injury and promoting ventilatory heterogeneity [5,13,26,27]. Sustained primarily by the diaphragm, this excessive load may also induce direct muscle injury, as demonstrated by animal models and morphometric analyses [8^▪▪^]. On the other hand, diaphragmatic dysfunction arises as a consequence of P-SILI: progressive lung injury and excessive mechanical burden lead to muscle fatigue, structural damage, and loss of contractile force, which in turn perpetuate respiratory failure and dependency on ventilatory support [8^▪▪^,^28^,^29^].

Although such reflections may appear theoretical, they help explain how clinical observations guide therapeutic strategies and deepen our understanding of pathophysiological mechanisms. Crucially, inspiratory effort can be modulated, not necessarily abolished. P-SILI is most likely when sedation is insufficient or ventilatory support inadequate, forcing patients to generate vigorous efforts. Sedation can influence both drive and effort, though its effects vary by agent and dose [30^▪^]. For example, propofol reduces respiratory drive (P0.1), effort (ΔPes), and dynamic lung-distending pressure, suggesting a protective effect, while opioids may blunt drive without consistently lowering effort [30^▪^]. Importantly, the impact of propofol follows a U-shaped curve: too little sedation permits excessive drive, favoring P-SILI, whereas deep sedation suppresses effort entirely, rendering the patient passive and vulnerable to VILI from excessive pressures and volumes.

A similar balance applies to ventilatory support or assistance. Insufficient support forces patients to generate vigorous, potentially injurious efforts. Conversely, excessive support renders them “quasi-passive,” with tidal volume determined primarily by compliance and ventilator settings rather than by patient effort. In this state, high driving pressures and nonprotective tidal volumes (>8 ml/kg) may arise despite minimal effort, exposing the lung to VILI [31,32].

Thus, the clinical challenge in preventing P-SILI is not merely to blunt inspiratory effort, but to shape it, preserving protective breathing while avoiding both unrestrained effort and injurious passivity.

### Central modulation of respiratory drive

Clinical evidence suggests that selectively targeting the diaphragm can effectively modulate respiratory drive and limit P-SILI. Bilateral phrenic nerve block has been shown to abolish excessive negative esophageal swings, halve dynamic transpulmonary pressures, and improve lung protection. Nakayama *et al.* [33] and Levis *et al.* [34] reported that continuous bilateral interscalene phrenic block using local anesthetics (lidocaine or mepivacaine) suppressed strong inspiratory efforts, reduced ΔPes and diaphragmatic electrical activity, and allowed patients to remain awake without prolonged neuromuscular blockade. These findings support the concept that selectively modulating diaphragmatic activity can interrupt the vicious cycle of P-SILI while minimizing ICU-acquired weakness.

Pharmacological central modulation is also under investigation. The ongoing DRIVE trial (NCT05514483) is testing ondansetron, a 5-HT_3_ antagonist, to determine whether attenuating chemoreceptor-mediated drive can reduce excessive inspiratory effort without deep sedation or paralysis [35].

Sedation remains the most established approach but it follows a U-shaped relationship. Light sedation fails to control drive, resulting in high ΔPes and dynamic strain, whereas deep sedation suppresses effort, predisposing patients to ventilator-induced overdistension and diaphragmatic atrophy. The LANDMARK trial highlighted that intermediate titration of propofol reduces P0.1, ΔPes, and dynamic distending pressures, achieving a balance where patient effort is controlled without complete passivity [30^▪^].

### Peripheral modulation of respiratory mechanics

Excessive inspiratory effort can directly injure the diaphragm through sarcomeric disruption and contractile fatigue, prolonging mechanical ventilation and worsening outcomes [36]. The effect of effort and end-expiratory pressure depends on lung compliance: stiff lungs amplify dorsal overdistension, while preserved compliance and oxygenation mitigate it [18^▪^,^37^].

Physiological studies clarify that pendelluft, although common during spontaneous breathing, is not the main driver of overdistension. Bello *et al.* [37] and Bassi *et al.* [38] showed that higher PEEP does not systematically reduce pendelluft, while overdistension persists. This indicates that injury is primarily determined by excessive inspiratory effort in the setting of reduced compliance and severe hypoxemia, with total lung-distending pressure being the key determinant of P-SILI rather than intrapulmonary gas shifts.

Early CPAP may counteract these mechanisms by stabilizing alveoli, increasing functional residual capacity, and limiting negative intrathoracic pressure swings that promote pulmonary edema [39]. In a 10-year cohort of 3898 infants with ARF, 752 treated with nasal bubble CPAP achieved an overall effectiveness of 96.5%, with 73% avoiding ICU transfer and most escalated patients avoiding intubation. Compared with historical controls, ICU admissions and invasive ventilation were significantly reduced, underscoring CPAP's dual role in improving oxygenation and attenuating injurious effort [39].

In adults, CPAP and NIV have shown consistent benefits for oxygenation. In patients with early COVID-19 hypoxemic respiratory failure, both interventions improved oxygenation compared to standard oxygen while keeping transpulmonary pressure within safe limits [40]. CPAP stabilizes alveoli through continuous PEEP and redistributes perfusion to better-ventilated regions; however, unlike NIV, it does not consistently unload inspiratory effort, and its effect on breathing workload varies across patients [41].

Prone positioning is another strategy to reduce overdistension. Slobod *et al.* [42] demonstrated that in unilateral lung injury, prone positioning reduces shunt and improves oxygenation by redistributing ventilation toward dorsal regions that receive substantial perfusion. Clinical evidence shows that prone positioning enhances alveolar recruitment, reduces collapse, and promotes a more uniform distribution of pulmonary stress and strain, ultimately improving V/Q matching and oxygenation [43,44].

Hypoxemia itself can amplify respiratory drive through chemical stimulation (chemoreceptors) or perceptual pathways (dyspnea disproportionate to gas exchange impairment). Studies of nasal high flow (NHF) suggest that even without major improvement in oxygenation, NHF reduces inspiratory drive (P0.1) and dyspnea, likely via trigeminal TRPM8 receptor activation in the nasal cavity [45^▪^]. This “sensory modulation” demonstrates that respiratory effort can be attenuated not only by correcting gas exchange but also by reshaping afferent perception, providing a novel approach to mitigating P-SILI.

### Preservation of diaphragm function

A key element to take into account while trying to avoid P-SILI is the preservation of diaphragmatic function, balancing effective effort with protection from overuse injury and disuse atrophy (ventilator-induced diaphragmatic dysfunction VIDD). In post-ICU COVID-19 patients, a 4-week inspiratory muscle training (IMT) program reduced biomarkers of muscle injury (CK-M, slow sTnI) and improved FEV_1_, FVC, maximal inspiratory pressure (PImax/PMmax), and grip strength, although effects on exercise tolerance and dyspnea were limited by short follow-up [46]. The rationale is improved motor-unit recruitment and neuromuscular coordination, which increases efficiency, shifts the diaphragm pressure–time curve, lowers ΔPes for a given minute ventilation, and promotes protective breathing.

In mechanically ventilated patients, percutaneous phrenic stimulation can reproduce physiological drive and prevent VIDD when sedation or high ventilatory assistance suppresses neural activity. Trials have shown feasibility and safety, with stable ventilator synchronization, protective WOB (0.2–2.0 J/l), and diaphragm thickening of +7.8% at 24 h and +15% at 48 h, indicating an atrophy preventing effect [47]. Automated on-demand systems can maintain a “minimum protective” level of diaphragmatic activity during controlled mechanical ventilation, preserving tone and neuro-ventilatory coupling without generating injurious ΔPes [48].

**FIGURE 2:**
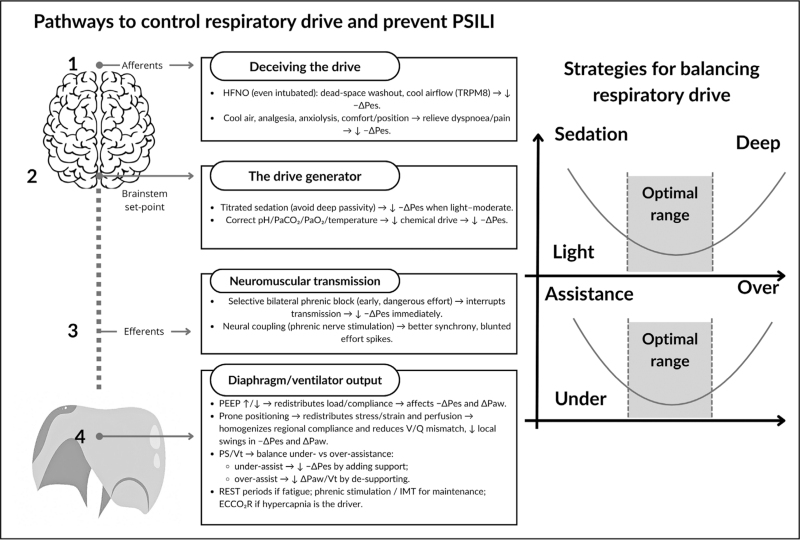
Pathways to modulate respiratory drive and prevent patient self-inflicted lung injury (P-SILI). Excessive inspiratory effort can be reduced at four levels: (1) afferent inputs (modulating sensory/comfort signals), (2) central drive, (3) neuromuscular transmission, and (4) the effector level (diaphragm–ventilator output). The right panel shows the U-shaped relationship between sedation/assistance and effort, with an “optimal range” between excessive drive and injurious passivity. EAdi, electrical activity of the diaphragm; ECCO_2_R, extracorporeal CO_2_ removal; IMT, inspiratory muscle training; paCO_2_, arterial CO_2_; paO_2_, arterial O_2_; PEEP, positive end-expiratory pressure; Vt, tidal volume; ΔPaw, airway pressure swing; ΔPes, esophageal pressure swing.

In ARDS/AHRF patients with high ventilatory ratios or refractory hypercapnia, extracorporeal CO_2_ removal (ECCO_2_R) can reduce chemical drive and inspiratory muscle load, allowing protective Vt and ΔP without abolishing diaphragm activity. Clinical reports show lower ΔPes and ΔPL,dyn, reduced sedation and inspiratory support, and improved diaphragm thickness, RSBI, and MIP/MEP [49] (Fig. 2).

## CONCLUSION

Overall, the literature clarifies the mechanisms of P-SILI and outlines potential therapeutic strategies. In clinical practice, the key challenge is timing interventions to preserve the “protective window” from the acute phase through weaning and rehabilitation. Modulation of respiratory drive or neuromuscular pathways is most effective in the acute phase, while diaphragmatic modulation supports recovery and rehabilitation. Current evidence, however, does not yet define the optimal duration or combination of these interventions.

## Acknowledgements

*None.*

### Financial support and sponsorship

*None.*

### Conflicts of interest

*There are no conflicts of interest.*

## REFERENCES AND RECOMMENDED READING

Papers of particular interest, published within the annual period of review, have been highlighted as:

▪ of special interest
▪▪ of outstanding interest
